# Giant paratesticular dedifferentiated liposarcoma with intraabdominal extension: a case report

**DOI:** 10.3389/fonc.2023.1216776

**Published:** 2023-07-26

**Authors:** Muhammad Hasif Azizi, Iqbal Hussain Rizuana, Yin Ping Wong, Khairiyah Sidek, Xeng Inn Fam

**Affiliations:** ^1^ Urology Unit, Department of Surgery, Faculty of Medicine, Hospital Canselor Tuanku Muhriz, Universiti Kebangsaan Malaysia, Kuala Lumpur, Malaysia; ^2^ Department of Radiology, Faculty of Medicine, Hospital Canselor Tuanku Muhriz, Universiti Kebangsaan Malaysia, Kuala Lumpur, Malaysia; ^3^ Department of Pathology, Faculty of Medicine, Hospital Canselor Tuanku Muhriz, Universiti Kebangsaan Malaysia, Kuala Lumpur, Malaysia; ^4^ Department of Oncology, Faculty of Medicine, Hospital Canselor Tuanku Muhriz, Universiti Kebangsaan Malaysia, Kuala Lumpur, Malaysia

**Keywords:** testicular mass, giant liposarcoma, giant paratesticular liposarcoma, paratesticular tumour, dedifferentiated liposarcoma, rhabdomyosarcoma, radical orchidectomy, laparoscopic

## Abstract

Giant paratesticular liposarcoma is a rare presentation of paratesticular tumor. We present a case of the largest paratesticular liposarcoma described to date with a weight of 4,100 g and measuring 460 × 210 × 130 mm. It was initially mistaken as an inguinoscrotal hernia until a contrast-enhanced computed tomography (CECT) scan of the abdomen and pelvis revealed a huge left paratesticular tumor extending from the scrotum to the mid-abdomen. The challenge was to achieve a tumor-free margin orchidectomy due to the poor fat plane of the tumor to the external iliac artery, psoas muscle, descending colon, and anterior abdominal wall. The surgery was started with laparoscopic dissection for the intraabdominal part of tumor from the vital structure, then followed by inguinal radical orchidectomy and inguinal mesh repair. Postoperative histopathological report revealed a paratesticular dedifferentiated liposarcoma with rhabdomyosarcomatous differentiation with clear margin. The patient had good recovery post operation.

## Introduction

Paratesticular liposarcoma is a rare malignant tumor originating from mesoderm. There are less than 200 cases reported in the English literature to date (1–3), with very few reported cases measuring more than 10 cm and were referred to as giant liposarcoma (2–4). It mostly occurs in the fifth to seventh decade of life. We report a case of a giant paratesticular liposarcoma with a weight of 4,100 g measuring 460 × 210 × 130 mm, which is the largest paratesticular liposarcoma reported in English literature. Owing to the rarity of the giant paratesticular liposarcoma, there is no universal consensus on the surgical approach of the tumor excision. This case highlights how tumor-free margin radical orchidectomy and high inguinal spermatic cord excision can be achieved by the laparoscopic-assisted approach for a giant paratesticular liposarcoma. Written informed consent was obtained from the patient for the publication of this case report and any accompanying images.

## Case report

This is a case of a 73-year-old man who presented to the urology clinic with left inguinoscrotal swelling for 3 years. The swelling originated in the scrotum and gradually increased in size, ascending towards the inguinal region. It was not accompanied by any pain. He did not have any other constitutional symptoms and no family history with malignancy. It was initially diagnosed as hernia by the general practitioner and the patient did not seek further intervention until the tumor had grown into a large mass and significantly impacting his quality of life.

**Figure 1:**
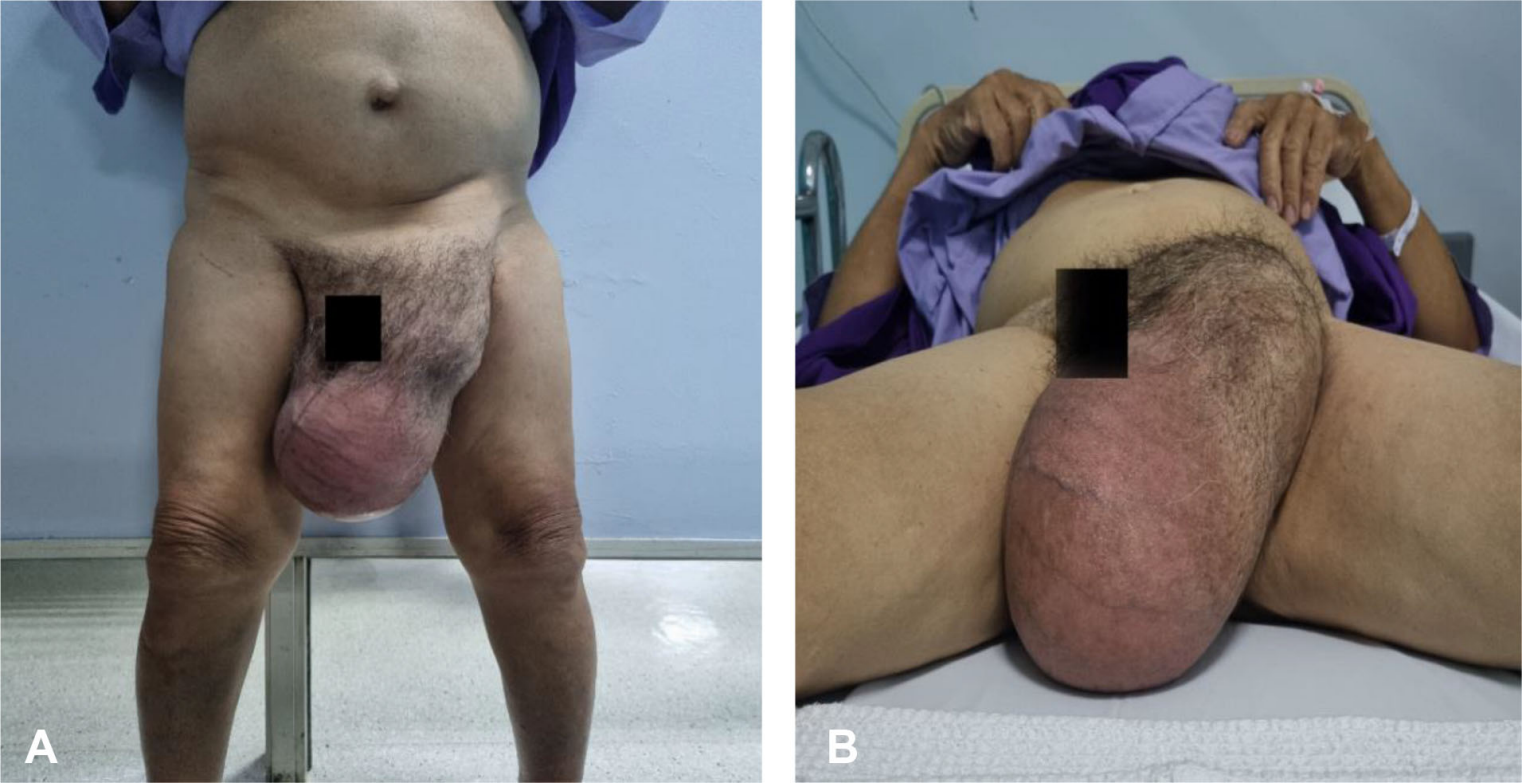
**(A, B)** Huge left inguinoscrotal testicular tumor. It was a hard mass, non-tender, non-pulsatile, and not expanding with a cough impulse.

**Figure 2:**
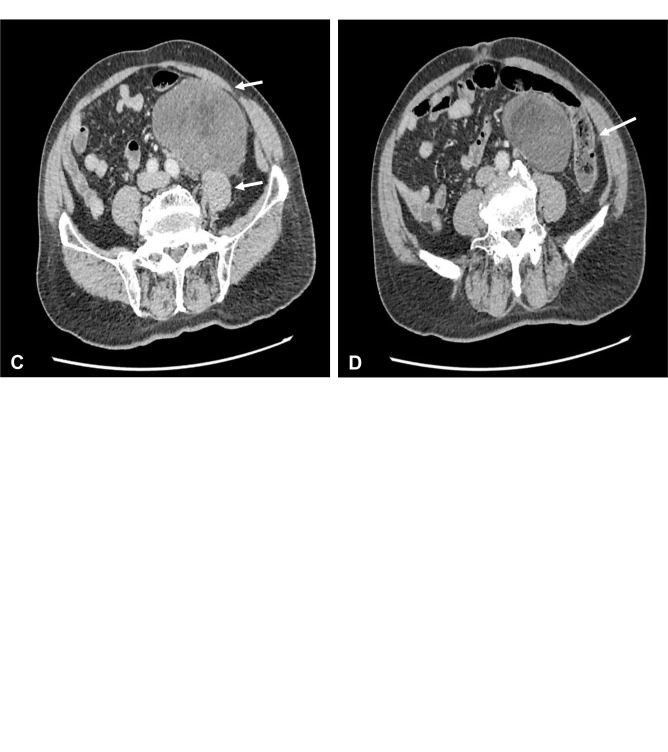
**(A)** Coronal view of contrast-enhanced computed tomography (CECT) scan showed huge left testicular tumor extending from scrotum until intraabdominal up to umbilical level with a length of 43 cm. **(B)** Coronal view of CECT scan showed the testicular tumor encasing the left external iliac artery. **(C)** Axial view of CECT scan showed close proximity to left psoas muscle and anterior abdominal wall. **(D)** Axial view of CECT scan showed close proximity to the descending colon.

On clinical examination, there was a huge inguinal scrotum tumor that was palpable up to the umbilical level, measuring approximately 50 × 20 × 10 cm (**Figure 1**). The tumor was hard, non-tender, non-pulsatile, and not expanding with cough impulse. Contrast-enhanced computed tomography (CECT) scan of the thorax, abdomen, and pelvis showed a huge well-encapsulated heterogeneous mass seen within the left scrotum extending to mid-abdomen measuring approximately 42.7 cm in length. It was in close proximity with anterior abdominal wall anteriorly as well as the left psoas and left external iliac artery posteriorly. There was no lymph node involvement and no distant metastasis reported (**Figure 2**). Serum beta-human chorionic gonadotropin (β-HCG) level was <2.3 mIU/ml and α**-**fetoprotein (AFP) was over 2.74 ng/ml, while serum lactate dehydrogenase (LDH) was 224 IU/L. The provisional diagnosis was left paratesticular tumor.

**Figure 3:**
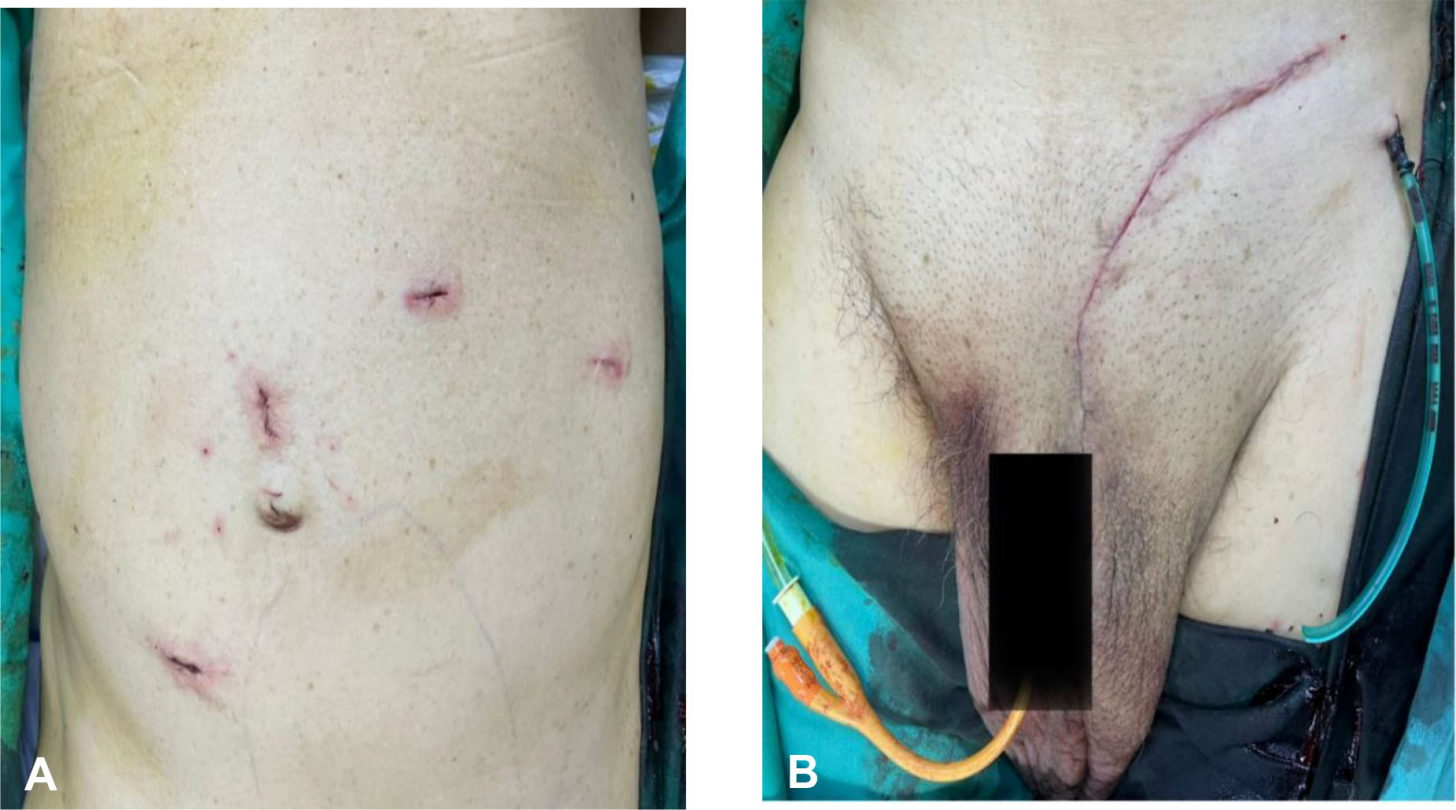
**(A)** Laparoscopic port incision; 2 × 12 mm camera ports were inserted at supraumbilical and right paramedian region and 2 × 5 mm working ports were inserted at the left upper quadrant. **(B)** Transinguinal incision for excision of the testicular tumor. Drain was inserted into the scrotum.

**Figure 4:**
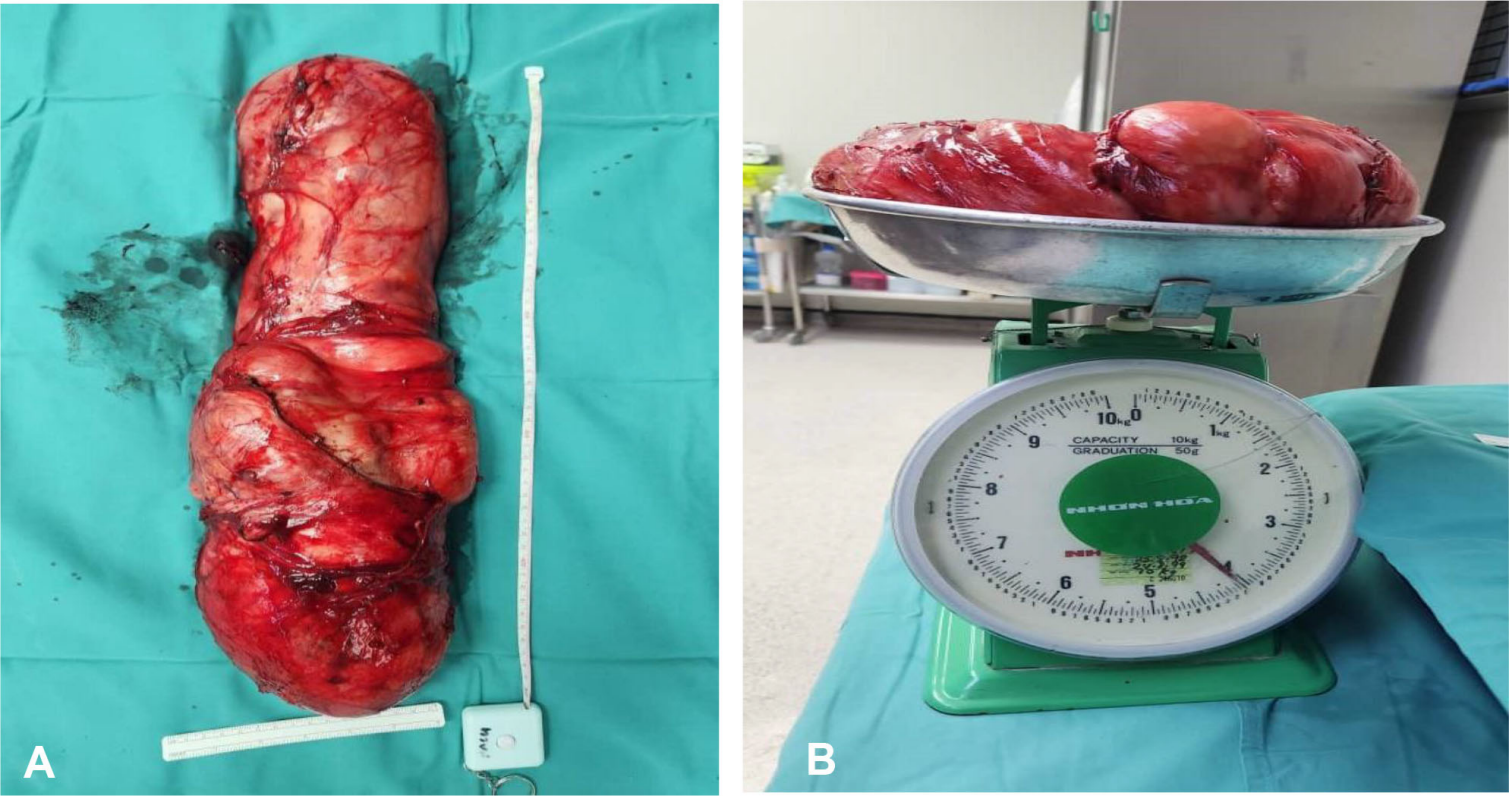
**(A)** Gross specimen of the left paratesticular tumor measured 50 × 20 × 10 cm. **(B)** The left testicular tumor weighed 4.1 kg on the weighing scale.

A multidisciplinary therapy discussion was held and decided for surgical excision. We successfully performed laparoscopic dissection and mobilization of the intraabdominal part of the tumor from the external iliac artery, descending colon, and anterior abdominal wall without causing any injuries to the mentioned structures. Then, we continued the surgery *via* inguinal incision to complete the tumor mobilization from the scrotum and delivered the tumor en bloc through inguinal incision (**Figure 3**). Inguinal wall was repaired with mesh. The tumor weighed 4,100 g (**Figure 4**), and it is the largest described to date in literature. The operation was performed under general anesthesia and lasted approximately 3 h. The operation resulted in minimal blood loss, estimated to be approximately 100 ml. The drain output was very minimal, and it was removed on day 2 post operation. Patient was discharged well. One month following the surgery, the patient visited the urology clinic where a fully healed wound was observed, and no presence of seroma was noted.

**Figure 5:**
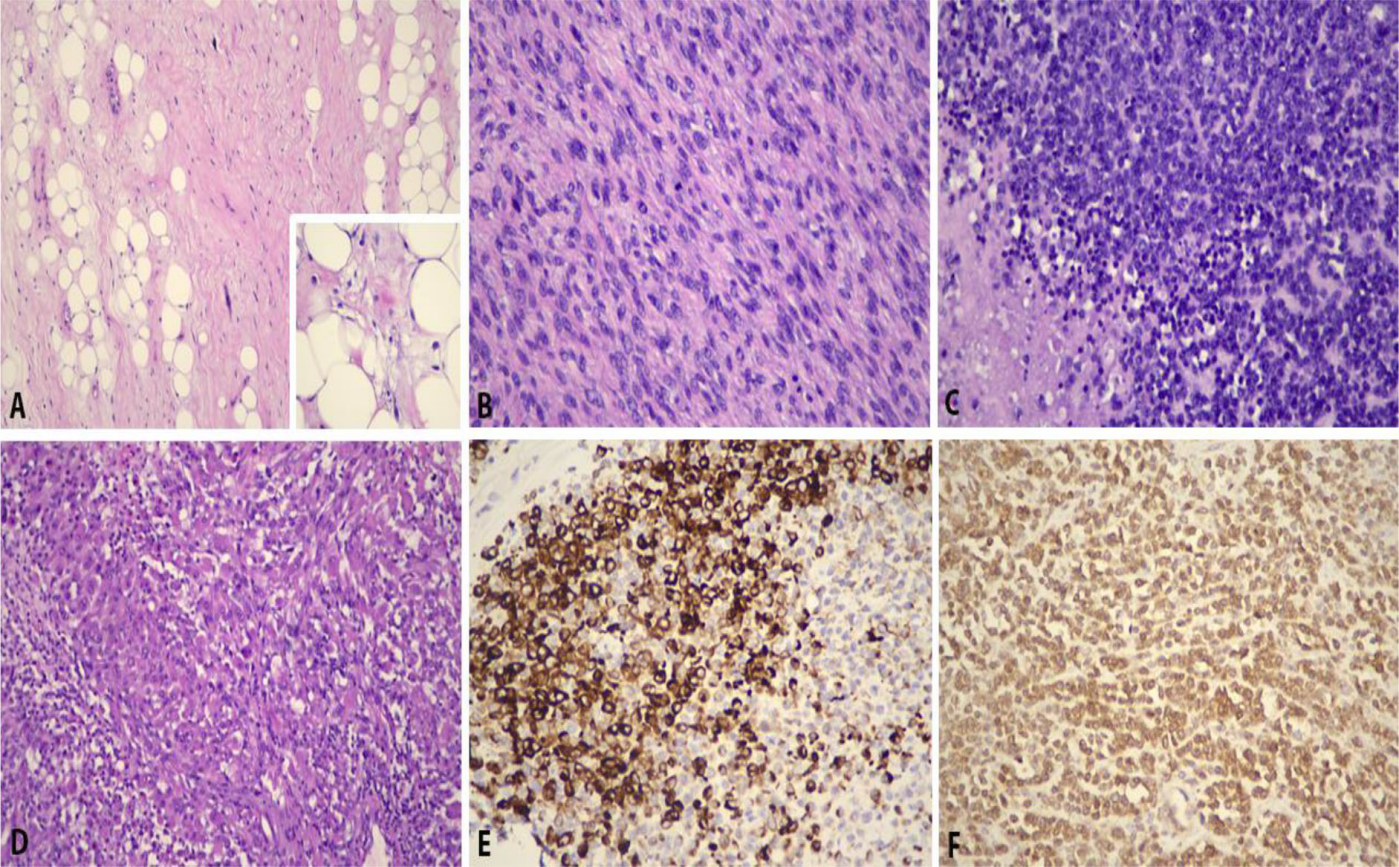
Histopathological features of paratesticular dedifferentiated liposarcoma with rhabdomyosarcomatous component. **(A)** Well-differentiated tumor showing atypical spindle cells (H&E, ×200). Inset shows lipoblasts (H&E, ×400). **(B)** Dedifferentiated tumor is cellular and resembles pleomorphic spindle cells with hyperchromatic nuclei and inconspicuous nucleoli (H&E, ×400). **(C)** In areas, small round blue malignant cells are seen (H&E, ×400). **(D)** Focal rhabdomyosarcomatous elements are observed (H&E, ×400), in which they are immunopositive for **(E)** desmin (desmin, ×400) and **(F)** myoD1 (myoD1, ×400).

Microscopically, the tumor exhibited high-grade sarcoma (estimated to approximately 40% of the entire tumor bulk) in addition to a background of classic well-differentiated liposarcoma. The high-grade sarcomatous component was cellular, demonstrating pleomorphic spindle cells with hyperchromatic nuclei and inconspicuous nucleoli; in areas, small round malignant cells were seen. Focal rhabdomyosarcomatous differentiation accounting for approximately 20% of the tumor was also observed (**Figure 5**). A diagnosis of a paratesticular dedifferentiated liposarcoma with rhabdomyosarcomatous differentiation was rendered. The surgical margins were clear from tumor infiltration, and there was no spermatic cord involvement.

Given the aggressive nature of this tumor, the patient was advised to undergo adjuvant chemotherapy. However, because of the patient’s advanced age and concerns regarding the potential side effects of chemotherapy, they firmly declined this treatment option.

**Figure 6:**
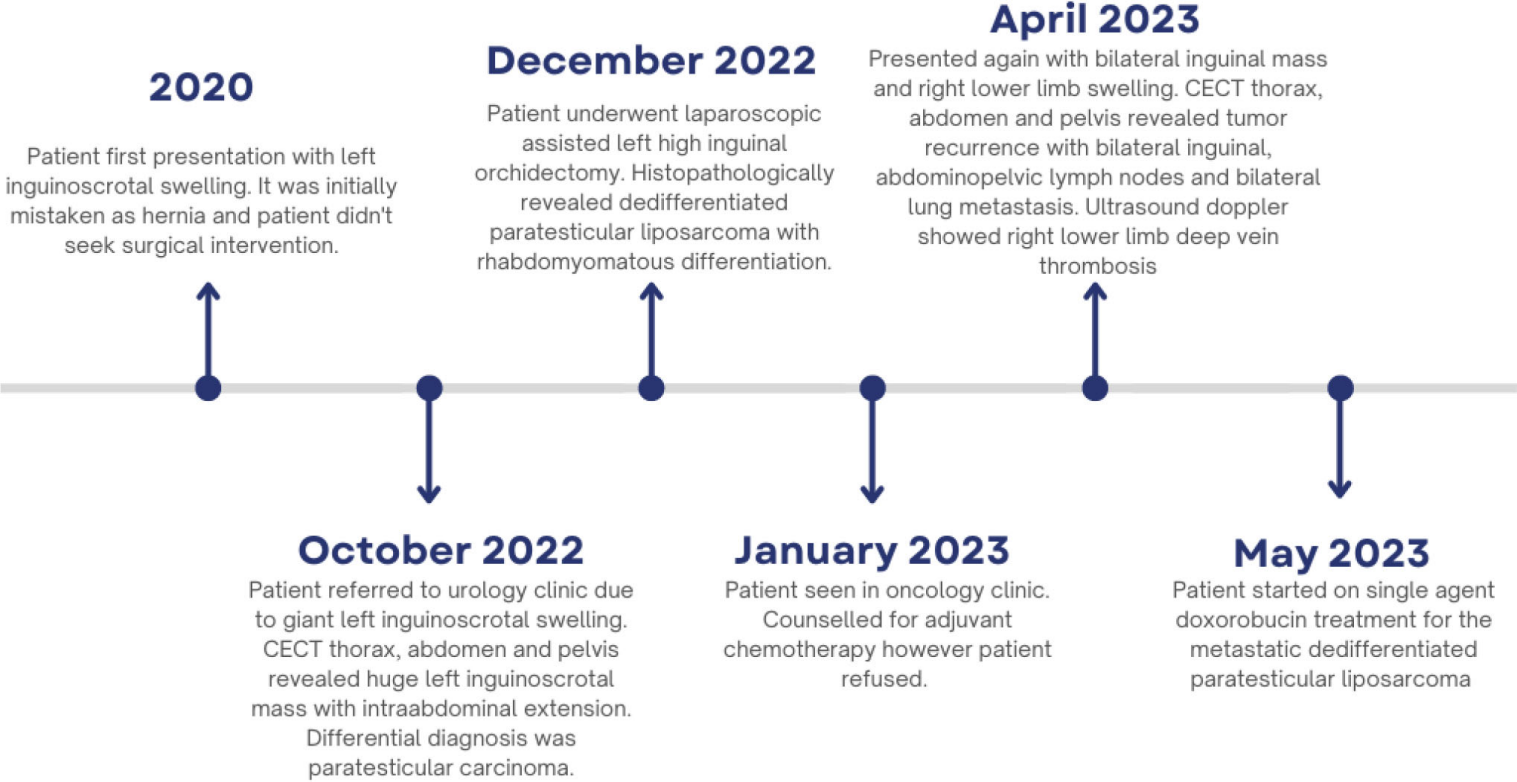
Disease timeline from its presentation to the treatment of care.

At the 3-month post-operation follow-up, the patient underwent CECT imaging of the thorax, abdomen, and pelvis for surveillance purposes. During the clinic visit, the patient expressed concern about bilateral inguinal masses and swelling in the right lower limb. Unfortunately, the CECT scan revealed a recurrence of a tumor in the left scrotal wall, as well as metastases to the bilateral inguinal and abdominopelvic lymph nodes and bilateral lungs. Additionally, an ultrasound Doppler examination detected a deep vein thrombosis (DVT) in the right lower limb. To address the DVT, the patient was prescribed oral rivaroxaban at a dosage of 15 mg twice daily. After extensive discussion, the patient agreed to undergo chemotherapy and is currently receiving single-agent doxorubicin treatment for the metastatic dedifferentiated paratesticular liposarcoma (**Figure 6**).

## Discussion

Liposarcoma is a type of soft tissue malignancy that arises embryologically from mesodermal tissue. It is the most common histopathological type of sarcoma (46%); the second most common is leiomyosarcoma (20%), followed by histiocytoma (13%) and rhabdomyosarcoma (9%). As a type of sarcoma, it can be presented at the head and neck region, extremities, and retroperitoneum. Seventy percent of the sarcomas arise from the extremities and retroperitoneum. Paratesticular liposarcoma, however, is a very uncommon condition with only less than 200 cases reported in the literature (1–3). Giant paratesticular liposarcoma is an even rarer presentation and was defined as a tumor size greater than 10 cm (2–4). It is a slow-progressing disease and usually affects men between the fifth decade and the seventh decade. Liposarcoma is the most common soft tissue sarcoma (5). According to the 2020 WHO classification of sarcoma (6, 7), there are five histopathological subtypes of liposarcoma, namely, well differentiated, dedifferentiated, myxoid, pleomorphic, and myxoid pleomorphic. The most common paratesticular liposarcoma is well-differentiated liposarcoma (WDL) and dedifferentiated liposarcoma (DDL) (7–9). Both types showed similar clinical presentation; however, the characteristic of the tumor is different. DDL is a more aggressive tumor; it carries a poor prognosis and a high possibility to metastasize compared to WDL. The local recurrence rate of DDL is 40% and the metastatic rate is 15%–30% (9).

Giant paratesticular liposarcoma usually presents in men over the age of 50 with slowly developing scrotal swelling and stiffness. Clinical diagnosis is challenging as the giant paratesticular liposarcoma is often mistakenly diagnosed as inguinoscrotal hernia or cord lipoma and hence the delay in diagnosis. Therefore, radiological imaging is needed to guide the diagnosis. Performing CECT scan or magnetic resonance imaging (MRI) can lead to a more specific diagnosis. Both CECT scan and MRI are the best imaging studies to detect any compression or invasion to the nearby organs. MRI, however, remains the gold standard investigation method for soft tissue tumor as it helps to characterize the lesion and delineate the degree of the local tumor invasion and also the tumor stage (10). In our case, MRI was not done as CT scan images had provided us sufficient information to proceed with surgery.

There is no “one size fits all” approach to the treatment of liposarcoma, particularly retroperitoneal sarcoma. Surgical resection has traditionally been the only potentially curative approach. It is best to achieve resection with microscopically negative margins (R0 resection) though it can be challenging as most of the time the tumor tends to be huge. The role of radiation therapy and chemotherapy, either given preoperatively or postoperatively, continues to be debated, and there is no consensus as to the best approach for all patients.

Radical orchidectomy and high inguinal spermatic cord excision remained the gold standard of treatment (11, 12). We chose to perform laparoscopic dissection and mobilization of the intraabdominal part of the tumor over laparotomy, and follow by dissection and mobilization of the scrotum part of the tumor through inguinal incision. The laparoscopic-assisted technique has successfully reduced the morbidity of this patient. The patient was able to ambulate on day 1 of the operation and was discharged home on day 2 of the operation.

The role of preoperative radiotherapy is controversial. There is no evidence-based guideline on the tumor size to consider neoadjuvant radiotherapy. Some institutions offer radiotherapy to large well-differentiated liposarcoma. Some argue that patients tend to die from local recurrence and not from distant metastasis unless in a dedifferentiated or high-grade tumor, which was shown in the randomized phase III STRASS trial (13). Preoperative radiotherapy was not offered in this case as the mass is so huge; it will irradiate a significant dose, causing a lot of organs at risk such as small bowel, kidney, bladder, rectum, and testes. Furthermore, the size of the tumor is considered astronomical for radiation technically; the maximum size of the radiation field for the LINAC machine is 40 cm. This tumor’s maximum diameter measures 50 cm.

Following resection of the retroperitoneal sarcoma without neoadjuvant therapy, adjuvant radiotherapy was not offered for a low-grade, completely resected (margin-negative) tumor. However, sometimes postoperative RT could be considered for patients with R0/R1 resected high- or intermediate-grade tumors that are at risk for local recurrence. In this case, we decided to just observe because it is not possible to deliver postoperative adjuvant radiotherapy to such a huge area with acceptable morbidity.

The benefit of adjuvant chemotherapy following surgical resection of a soft tissue sarcoma is controversial. An updated meta-analysis suggested that the use of an optimal adequately dosed anthracycline/ifosfamide-containing regimen significantly prolongs survival, but the analysis did not include the two largest trials, both conducted in Europe and both testing the value of an anthracycline- and ifosfamide-containing regimen (14). A pooled analysis of both trials indicated no benefit from this approach (15). Approximately 10% of the patients entered into these trials had central tumors.

## Conclusion

We highlight a challenging case of radical orchidectomy with high inguinal spermatic cord excision *via* a laparoscopic-assisted approach for the largest paratesticular liposarcoma described to date. The giant paratesticular liposarcoma is a rare condition and often misdiagnosed as inguinoscrotal hernia or other benign scrotal swelling, resulting in a delay in diagnosis. Margin-negative excision remains the standard of treatment.

## Data availability statement

The original contributions presented in the study are included in the article/supplementary material. Further inquiries can be directed to the corresponding author.

## Ethics statement

Ethical review and approval was not required for the study on human participants in accordance with the local legislation and institutional requirements. The patients/participants provided their written informed consent to participate in this study. Written informed consent was obtained from the individual(s) for the publication of any potentially identifiable images or data included in this article. Written informed consent was obtained from the participant/patient(s) for the publication of this case report.

## Author contributions

MA prepared the draft. XF conceptualized the article and was the primary surgeon. XF and IR edited and finalized the draft. YW, IR, and KS prepared the images, photos, and captions. All authors contributed to the article and approved the submitted version.
